# The Double-Edged Effects of MLN4924: Rethinking Anti-Cancer Drugs Targeting the Neddylation Pathway

**DOI:** 10.3390/biom14070738

**Published:** 2024-06-21

**Authors:** Haoming Tang, Xin Pang, Shun Li, Liling Tang

**Affiliations:** 1 Key Laboratory of Biorheological Science and Technology, Ministry of Education, College of Bioengineering, Chongqing University, Chongqing 400044, China; 202219021021@stu.cqu.edu.cn (H.T.); 202319131144@stu.cqu.edu.cn (X.P.); 2 Department of Immunology, School of Basic Medical Sciences, Chengdu Medical College, Chengdu 610500, China; 3 Department of Spine Surgery, People’s Hospital of Longhua, Affiliated Hospital of Southern Medical University, Shenzhen 518109, China

**Keywords:** MLN4924, cancer, neddylation pathway, double-edged effect

## Abstract

(1) Background: The neddylation pathway assumes a pivotal role in the initiation and progression of cancer. MLN4924, a potent small-molecule inhibitor of the NEDD8-activating enzyme (NAE), effectively intervenes in the early stages of the neddylation pathway. By instigating diverse cellular responses, such as senescence and apoptosis in cancer cells, MLN4924 also exerts regulatory effects on non-malignant cells within the tumor microenvironment (TME) and tumor virus-infected cells, thereby impeding the onset of tumors. Consequently, MLN4924 has been widely acknowledged as a potent anti-cancer drug. (2) Recent findings: Nevertheless, recent findings have illuminated additional facets of the neddylation pathway, revealing its active involvement in various biological processes detrimental to the survival of cancer cells. This newfound understanding underscores the dual role of MLN4924 in tumor therapy, characterized by both anti-cancer and pro-cancer effects. This dichotomy is herein referred to as the “double-edged effects” of MLN4924. This paper delves into the intricate relationship between the neddylation pathway and cancer, offering a mechanistic exploration and analysis of the causes underlying the double-edged effects of MLN4924—specifically, the accumulation of pro-cancer neddylation substrates. (3) Perspectives: Here, the objective is to furnish theoretical support and novel insights that can guide the development of next-generation anti-cancer drugs targeting the neddylation pathway.

## 1. Introduction

Neddylation is a type of post-translational modification (PTM). This process involves the covalent attachment of the neural precursor cell expressed developmentally downregulated protein 8 (NEDD8) to the substrate protein through a series of enzymatic reactions similar to ubiquitination [1,2,3,4,5,6]. After binding to a conservative lysine target, NEDD8 can affect substrate conformation, stability, localization, and functions [2,7,8,9,10,11].

Cullin–RING ligases (CRLs) are the largest family of E3 ubiquitin ligases and classic neddylation substrates. Structurally, CRL is a multi-protein complex. After undergoing the necessary neddylation of its Cullin subunit, it can mediate the degradation of 20% intracellular proteins via the ubiquitin–proteasome system (UPS) [1,4,12]. In addition, CRL-mediated ubiquitination can either activate, inhibit, or relocate the target proteins [4]. 

The neddylation pathway is a significant contributor to the development and progression of cancer. Therefore, the neddylation pathway is generally overexpressed in different types of cancer [7,9,13]. The causation includes but is not limited to the fact that the neddylation pathway can degrade tumor suppressors and accumulate oncoproteins, promoting cancer cell survival and proliferation [1,3]. Additionally, it was also demonstrated to be crucial to the regulation of the TME and tumor viruses [14,15]. Therefore, the neddylation pathway has emerged as a promising target for cancer treatment [1,3].

MLN4924/TAK-924/Pevonedistat was first discovered in 2009. It can react with NEDD8 to form a covalent adduct and block the initial stage of the neddylation pathway, eliciting the wide inactivation of neddylation substrates [1,16]. Therefore, MLN4924 can induce both the accumulation of anti-cancer factors and the instability of pro-cancer factors. This triggers multiple cellular responses against cancer, including DNA damage, cell cycle arrest, apoptosis, senescence, and autophagy [1,3]. For the TME, on which cancer cells depend, it was verified that MLN4924 directly targeting non-malignant cells within the TME, such as cancer-associated fibroblasts (CAFs), cancer-associated endothelial cells (CAEs), and tumor-associated macrophages (TAMs), can prevent cancer cells from leaving the niche by inhibiting tumor angiogenesis and immunosuppressive microenvironment [17,18,19]. In addition, MLN4924 is also characterized by its extensive antiviral properties [13]. Herein, tumor viruses are defined as viruses that are capable of initiating or promoting cancer. This means that the anti-cancer activity of MLN4924 can be exhibited indirectly through its antiviral effects [13,20]. In light of its great anti-cancer capabilities, MLN4924 has been under a series of I/II/III clinical trials for solid and non-solid cancers https://clinicaltrials.gov/search?cond=Cancer&intr=MLN4924 (accessed on 10 June 2024). 

Surprisingly, an increasing number of studies have offered proof that MLN4924 not only inhibits cancer development and progression but also promotes cancer cell survival, proliferation, and immune escape [21,22,23,24], significantly compromising its reputation as an anti-cancer drug. However, little research has focused on the dark side of MLN4924, and there is a lack of systematic evaluation of its pro-cancer effects. In this review, we provide a summary of the correlations between the neddylation pathway and cancer in addition to discussing the double-edged effects of MLN4924 and analyzing their mechanisms. Finally, we propose improved schemes for alleviating its side effects.

## 2. Neddylation Pathway and Cancer

The neddylation pathway is an attractive target for anti-cancer therapy due to its crucial role in regulating cancer cells, the tumor microenvironment, and tumor virus-infected cells [11,12,13]. However, the regulation of the neddylation pathway is biphasic, ultimately leading to the double-edged effects of MLN4924 [1,14,25].

### 2.1. Neddylation Substrates

**Figure 1:**
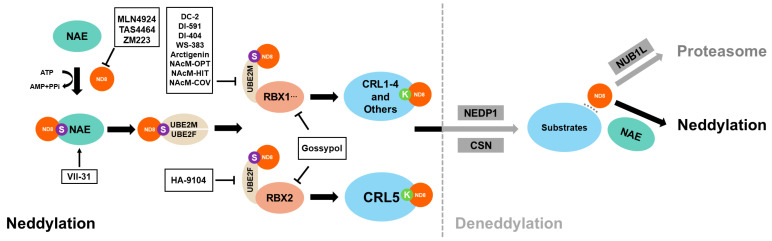
Scheme of enzymatic cascade reaction of neddylation and deneddylation. Neddylation begins with the binding of NAE and NEDD8, followed by the transfer of NEDD8 to E3 ligases, such as RBX1, RBX2, and DCN1, via UBE2M or UBE2F. Finally, NEDD8 covalently attaches to the conserved lysine residue of the substrates. Deneddylases, such as CNS5 and NEDP1, can mediate the deconjugation of NEDD8 from the substrates. The free NEDD8 can then be captured by NUB1L for lysosomal degradation or enter the next neddylation process under NAE guidance. So far, inhibitors targeting every step of the neddylation cascade have been developed. In addition, VII-31 can be used to activate neddylation.

Three enzymes are responsible for adding NEDD8 during the neddylation process. In order, they are the NAE (also known as NAE1-UBA3), the E2NED8-conjugating enzyme (UBE2M/UBC12 or UBE2F), and the NEDD8-E3 ligases [1,3]. The linkage of NEDD8 with substrates affects the activity, stability, function, and cell localization of the latter (Figure 1) [2,7,8,9,10,11]. Therefore, neddylated substrates participate in a series of essential biological processes, including transcription, autophagy, and signaling transduction [3,6,12].

It has been demonstrated that neddylation blockage can inhibit the downstream signaling pathways involved in the development of various diseases, including malignancies, particularly NF-κB signaling pathways [4,26]. Aside from classical substrates and CRLs, many other proteins have been identified as neddylation substrates and exhibit pro-cancer effects on different types of cancers (Table 1) [3,5]. For example, human epidermal growth factor receptor 2 (HER2), correlated with poor prognosis in breast cancer, was found to accelerate cancer progression in a NEDD8-dependent manner [8]. Olaizola et al. [4] demonstrated that NEDD8-conjugated proteins upregulated in cholangiocarcinoma (CCA) were involved in regulating the cell cycle, DNA repair, proliferation, and tumor-related immunosuppression. Noteworthy, many anti-cancer factors such as p53, p21, and PTEN can also pattern with NEDD8 and play an important role in the DNA damage response (DDR) of cancer cells [3,4,7]. However, the neddylation of these anti-cancer factors partially reverses their anti-cancer properties and instead promotes cancer development [7,27]. 

As mentioned above, numerous anti-cancer factors and pro-cancer factors are directly modified by NEDD8 [3], and certain neddylation substrates such as CRL, EGR1, and HIF-1α can simultaneously regulate both types of factors [5,12,28,29]. Therefore, the neddylation pathway can inhibit or promote cancer cell proliferation and invasion. This duality explains why MLN4924, a neddylation inhibitor, can have unintended pro-cancer side effects.

**Table 1:** Neddylation substrates.

Neddylation Substrates	Involvement in Biological Processes	Neddylation Effects	References
CRLs	Degrading anti-cancer factors	Promoting its activity	[1]
NIK	Inducing a non-canonical NF-κB pathway and accentuating inflammation	Promoting its ubiquitination and inhibiting its aberrant activation	[6]
PTEN	Inhibiting cancers via the PIK/FAK/MAPK signaling pathway	Reversing its function as a tumor suppressor	[7]
HER2	Enhancing breast cancer cell proliferation, survival, migration, and polarity changes	Promoting its accumulation	[8]
IRF7	Promoting host antiviral innate immunity against viruses	Promoting its nuclear translocation and preventing its dimerization with IRF5	[11]
LANA	Repressing ORF50p and the onset of KSHV lytic reactivation in primary effusion lymphoma	Promoting its activity	[25]
p53	Preventing the multiplication of damaged and potentially pre-cancerous cells	Inhibiting its transcriptional activity and function	[27]
SPH2	Binding to the ITIM of SIRPα and promoting macrophage phagocytosis of cancer cells	Inhibiting its linkage with ITIM	[30,31]
β-catenin	Playing a crucial role in the Wnt signaling pathway and promoting cancer cell migration and adhesion	Promoting its fast degradation	[32]
Coro1a	Promoting the recruitment of Rab7 to multivesicular bodies to reduce extracellular vesicle secretion	Promoting its activity	[33]
mGlu7	Modulating the maturation of excitatory presynaptic terminals	Promoting its ubiquitination and stabilizing its expression	[34]
TAK1	Mediating the signaling transduction induced by TGF beta and morphogenetic protein	Promoting its nuclear import	[35]
Gadd45a	Responding to environmental stresses by activating the p38/JNK pathway via MTK1/MEKK4 kinase	Promoting its nuclear export	[35]
Cofilin	Influencing neuron growth and cell migration during brain development	Its site-specific neddylation modulates cytoskeletal actin dynamics and neuron development	[36]
c-Src	Promoting cancer progression by activating the PI3K-AKT pathway	Promoting its polyubiquitination and degradation	[37]
PCNA	Assisting DNA polymerase in mediating DNA replication	Antagonizing its ubiquitination and inhibiting its interaction with polη	[38]
MyD88	Playing a central role in pro-cancer inflammation	Antagonizing its ubiquitination, reducing its dimerization, and suppressing NF-κB activity	[39]
MKK7	Impeding breast cancer proliferation and EMT phenotype via JNK phosphorylation	Inhibiting its basal kinase activity	[40]
CXCR5	Stimulating cell migration and motility	Targeting its location to the cell membrane	[41]
E2F-1	Mediating the G1-to-S-phase transition	Downregulating its stability and transcriptional activity	[42]

### 2.2. The Neddylation Pathways in Cancer Cells

The neddylation pathway is commonly overexpressed in various human cancers [43,44,45]. This pathway not only inhibits and/or reverses the functions of tumor suppressors but also promotes the resistance of cancer cells to chemotherapy [44], radiotherapy [46], and immunotherapy [4,45].

The neddylation substrates, especially CRLs, actively participate in key processes such as DNA damage repair and cell-cycle checkpoint signaling in DDR, which develop the resistance of cancer cells to chemotherapy and radiotherapy [4,44].

The neddylation pathways in cancer cells are also involved in transforming the microenvironment to favor cancer cells, impeding the activation of anti-cancer immunity [4,45]. The remodeling of the tumor microenvironment is partially caused in cancer cells by the cooperation between the neddylation pathway and the NF-κB signaling pathway. Mechanistically, the neddylation pathway in cancer cells overactivates the downstream NF-κB signaling pathway, resulting in chronic inflammatory reactions [47]. In the organism, NF-κB can be activated and translocated via canonical and non-canonical pathways, both of which may lead to tumor onset and require β-transducing repeat-containing protein (β-TrCP) participation [26,48]. After being assembled into CRL1 and undergoing the necessary NEDD8 modification, β-TrCP can mediate the degradation of the NF-κB inhibitor Iκ-B or the processing of p100, eventually leading to the nuclear translocation of NF-κB [49]. It has been demonstrated that the unrestricted activation of NF-κB can have a negative impact on anti-cancer immune responses: NF-κB activation through the neddylation pathway in lung cancer cells can induce the infiltration of TAMs and myeloid-derived suppressor cells (MDCSs) in chemotactic cytochrome ligand2 (CCL2)-dependent [49] and C-X-C motif chemokine 6 (CXCL6)-dependent [45] ways to alter a microenvironment that is supportive of cancer growth and progression.

In brief, cancer cells can transform the TME via secreting neddylation-NF-κB pathway substrates. This transformation commonly generates an immunosuppressive microenvironment that hinders the progress of anti-cancer immune responses.

### 2.3. The Neddylation Pathways in TME

The entire process, from tumor initiation to metastasis, occurs within a comfortable microenvironment. The TME is a dynamic environment surrounding cancer or cancer stem cells (CSCs). It consists of non-malignant cells, including immune cells (such as T cells, dendritic cells, and macrophages) and stromal cells (such as fibroblasts and vascular endothelial cells), and the extracellular matrix (ECM), containing collagen, laminin, and various soluble cell factors [14]. The communication between cancer cells and the TME is compact and partly mediated by the neddylation pathway. On the one hand, cancer cells can release signaling factors that recruit and remodel normal cells via the neddylation pathway [45,50]. On the other hand, within the TME, the neddylation pathway in non-malignant cells can directly regulate the biological behaviors of adjacent cancer cells, thus affecting their survival and proliferation [14,30]. In short, the TME is often associated with the advancement of cancer. 

In addition to cancer cells, recent research has verified that the neddylation pathway in non-malignant cells can reprogram the TME and significantly affect cancer progression by regulating their functions [14,19]. For instance, by targeting blockages in the neddylation pathway of CAFs, which are known to promote cancer cell proliferation, drug resistance, invasion, and migration [51], the proliferation and migration of CAFs can be inhibited and the secretion of pro-cancer factors can be reduced [14]. For macrophages, their phagocytosis/clearance of cancer cells was recovered with the neddylation of src homology region 2-containing protein tyrosine phosphatase 2 (SPH2), while the deneddylation of SPH2 abolished it [30]. The neddylation pathway also promotes the proliferation of vascular endothelial cells (VECs) [18,19], suggesting its involvement in tumor angiogenesis. Furthermore, the neddylation pathway, which regulates the maturation and activation of immune cells such as T cells and dendritic cells (DCs), plays a significant role in antitumor immunity [3,14,52].

### 2.4. Neddylation Pathways and Tumor Viruses

Cancer initiation is a complex process involving multiple steps and factors. Viral infection is one of the most significant factors contributing to carcinogenesis [53]. Herein, tumor viruses are defined as viruses that have pro-cancer capabilities. The acknowledged tumor viruses include hepatitis B virus (HBV), human papilloma virus (HPV), Epstein–Barr virus (EBV), human immunodeficiency virus (HIV), and Kaposi’s sarcoma-associated herpesvirus (KSHV) [53,54,55].

Recent research has revealed that the neddylation pathway profoundly affected the infection of tumor viruses [15]. For example, Zhao et al. [11] reported that neddylated IRF7 in myeloid cells could promote its nuclear translocation and host innate immunity against RNA viruses, indicating that MLN4924 treatment might abrogate this process. Chang et al. [25] reported that the KSHV latency-associated nuclear antigen (LANA) undergoes neddylation, which inhibits the latent-to-lytic switch in host cells. Likewise, the activation of the EBV latent-to-lytic switch required the presence of HIF-1α [56]. The neddylation pathway can accelerate the degradation of HIF-1α via UPS, inhibiting the reactivation of EBV [56]. This ultimately leads to the failure of lytic induction therapy for EBV+ cancer patients [56]. For HBV, the neddylation pathway can maintain HBV cccDNA transcription and promote HBsAg synthesis and release [13,20]. High levels of HBV cccDNA and HBsAg may lead to secondary liver diseases such as cirrhosis and hepatocellular carcinoma [13]. In addition, the neddylation pathway can promote HIV infection by allowing HIV-1 to hijack CRL5 via the viral protein Vif and degrade members of the APOBEC anti-HIV family via UPS [57]. Therefore, the neddylation pathway enables HIV-1 to break through the defenses of host cells to replicate. On the other hand, HIV-2 can recruit the antiviral factor SAM domain- and HD domain-containing protein 1 (SAMHD1) to CRL4 with the assistance of the HIV-2 viral protein X (Vpx). Once recruited, the neddylation–UPS pathway can potentiate the degradation of SAMHD1 and relieve its inhibition of SAMHD1 on the reverse transcription of HIV-2 [2,57,58].

## 3. Anti-Cancer Effects of MLN4924

Cancer cells can enhance their survival and growth as well as continuously recruit and reprogram normal cells within the microenvironment via the neddylation pathway, which is crucial to cancer cell dissemination. Hence, the neddylation pathway is frequently upregulated in cancer cells [3]. Non-malignant cells within the TME also regulate their functions to favor cancer cells via neddylation pathways [14]. In addition, tumor viruses can hijack host neddylation pathways to promote tumor-associated disease aggravation [13,15]. 

MLN4924, structurally similar to AMP and involved in NEDD8 activation, can inhibit the NAE by forming an adduct with NEDD8, thus blocking subsequent neddylation enzymatic reactions [16]. Therefore, MLN4924 can potently block the neddylation pathway in cancer cells, the TME, and tumor virus-infected cells to display its effective anti-cancer capacities [1,4,14]. MLN4924 targeting TME and tumor virus-infected cells expands its advantages as an anti-cancer drug [13,20,25,57].

### 3.1. MLN4924 Inhibits Cancer Cell Proliferation by Accumulating Cancer Suppressors

As an anti-cancer drug, MLN4924 has certain cytotoxic effects derived from the inhibition of neddylation, which is essential for maintaining normal cell functions [6,8,44]. However, due to the overexpression of the neddylation pathway in cancer cells, MLN4924 has confirmed specificity to distinguish cancer from normal tissue [1,13,59].

MLN4924 can induce the inactivation of CRLs, leading to the accumulation of CRL substrates. This buildup is associated with various cellular responses such as DNA damage, cell cycle arrest, apoptosis, senescence, and autophagy [1,12]. It should be noted that depending on the type of cancer cells, the effectiveness of MLN4924 as an anti-cancer agent may vary, suggesting its cell-specific cytotoxicity. 

Specifically, in cancer cells, MLN4924 can induce DNA re-replication stress and DDR via accumulating CRL substrates to activate cell-cycle checkpoints, resulting in cell cycle arrest and directly lowering the proliferation and growth of cancer cells [12]. Then, the senescence and/or apoptosis of cancer cells may be triggered to further enhance inhibitory effects [3]. In addition, by inhibiting the activity of mammalian target of rapamycin (mTOR), which is a negative regulatory factor of autophagy, or by promoting the production of reactive oxygen species (ROS), MLN4924 can induce autophagy in cancer cells. This, in turn, triggers the senescence and apoptosis of cancer cells [60,61,62,63]. Therefore, MLN4924 has been widely used to enhance the efficiency of chemotherapy and radiotherapy in treatment-resistant cells [64].

### 3.2. MLN4924 Represses the Reprogramming of the TME Mediated by Cancer Cells

In light of its multidimensional inhibition of the pro-cancer TME, research has been conducted on the significance of the neddylation pathway in reprogramming the TME with the help of MLN4924 [14].

On the one hand, MLN4924 can directly inhibit the formation of a pro-cancer microenvironment by targeting the non-malignant cells within the TME. For example, MLN4924 can stabilize glioma-associated oncogene homolog 1 (Gli1), which was identified as a substrate of CRL1 or CRL3 [65]. The accumulation of Gli1 in pancreatic stellate cells (PSCs), which are crucial stromal cells in the pancreatic cancer microenvironment, can restrict access to cancer cell migration [18]. However, the high level of Gli in pancreatic ductal adenocarcinoma cells (PDACs) seems adverse to their migration [66], indicating that targeting MLN4924 in PSCs and PDACs probably has opposing effects on cancer progression. For tumor-infiltrating lymphocytes (TILs), MLN4924 could target CD8+ T cells and enhance their cytotoxicity by promoting TNF-α and IFN-γ expression [17]. In addition to promoting the anti-cancer immunity mediated by cytotoxic T cells, MLN4924 can also inhibit immunosuppression involving regulatory T cells (Tregs) by abrogating their expansion and differentiation [52,67]. For non-immune cells within the TME, MLN4924 can deprive cancer cells of their metastatic capacity by restricting the neddylation-mediated pro-cancer functions of stromal cells such as CAFs and VECs [18,19]. 

On the other hand, due to the frequent interaction between cancer cells and their niche, cancer cells can secrete neddylation substrates to transform the TME, further facilitating their proliferation and spread [45]. MLN4924 can impede the communication between cancer cells and the surrounding non-malignant cells by blocking the neddylation pathway in cancer cells [45,62]. For example, Olaizola et al. [4] found that MLN4924 could reduce the viability of CAFs and halt tumor angiogenesis by regulating the secretome of CCA. In addition, targeting MLN4924 in uveal melanoma cells (UMs) can reduce the paracrine secretion of vascular endothelial growth factor-C (VEGF-C) in an NF-κB-dependent manner [62]. This inhibition can prevent angiogenesis and subsequent liver metastasis, which is a leading cause of UM mortality [62], suggesting that the cooperation between the neddylation and NF-κB pathways may be the key mechanism of the formation of immunosuppressive microenvironments. Undifferentiated, Zhou et al. [45] reported that MLN4924 can inhibit the neddylation–NF-κB-CXCL6 axis in lung adenocarcinoma cells (LUADs), which in turn prevents the recruitment of MDCSs. This further indicates that the NF-κB pathway plays a vital role in the development of the neddylation-mediated pro-cancer TME.

### 3.3. MLN4924 Delays the Aggravation of Diseases Mediated by Tumor Viruses

The neddylation pathway is involved in modulating innate immune response after viral infections to promote viral reproduction, in which NF-κB might cooperate, suggesting that many viruses are MLN4924-susceptible [15]. The suppressive capacities of MLN4924 on tumor viruses give it an additional advantage as an anti-cancer drug. Here, we present the antiviral mechanisms of MLN4924 against several tumor viruses.

HBVs can maintain and amplify the viral genome in two ways, potentially leading to hepatocirrhosis and hepatocellular carcinoma (HCC). Mechanistically, on the one hand, after HBV infection, the HBV X protein (HBx) can take over the CRL4 of host cells [20,68]. This enhances the degradation of SMC5/6 via UPS which helps to relieve the host restrictions on the replication of HBV cccDNA and the transcription of HBV RNA [20,68]. Therefore, abrogating CRL4 neddylation can recover host antiviral defense, indicating the direct anti-HBV activity of MLN4924. On the other hand, MLN4924 can activate ERKs by triggering EGFR dimerization, which was defined as an off-target effect of MLN4924 [13]. Thus, MLN4924 can inhibit the expression of ERK downstream transcription factors, such as C/EBPα, HNF-4α, and HNF-1α, which are required for HBV replication [13,22]. This inhibition leads to a reduction in the synthesis of HBV DNA and HBsAg [20]. Apparently, the two mechanisms mentioned above make MLN4924 have strongly suppressive effects on HBV, contributing it to being an ideal anti-HBV drug. In addition, given the significant dependence of HIV infection on the neddylation pathway, MLN4924 also exhibits inhibitory activity against HIV-1 and HIV-2 replication [57].

Lytic induction therapy is a rising treatment method for virus-associated cancer. It can specifically target and kill virus-positive cancer cells while clearing the viruses. The latent-to-lytic transition of EBV requires the presence of HIF-1α, which is a well-defined CRL2 substrate [69]. HIF-1α is maintained at low intracellular levels in EBV+ cancer cells due to the overexpressed neddylation pathway, which leads to the failure of lytic induction therapy. However, Kraus et al. [56] discovered that MLN4924 was a stabilizer of HIF-1α, which can activate the latent-to-lytic switch and induce EBV reactivation. Noteworthily, they also pointed out that in addition to HIF-1α, the correct phosphorylation of wild-type p53 in EBV+ cancer cells also to a great extent dictated the efficiency of lytic induction therapy [56]. The regulation of the tumor suppressor p53 is complicated and diverse. The possible modifications of p53 include phosphorylation, ubiquitination, sumoylation, neddylation, acetylation, methylation, O-GlcNAcylation, ADP-ribosylation, hydroxylation, and β-hydroxybutyrylation [70]. It was demonstrated that neddylation could inhibit the transcriptional activity of p53, whereas phosphorylation activated it [27]. The antagonism between neddylation and phosphorylation for p53 suggests that MLN4924 probably prevents p53 frombeing neddylated, thereby enhancing lytic induction therapy, which relies on the phosphorylation of p53.

### 3.4. MLN4924 Resensitizes Cancer Cells to Anti-Cancer Treatments

MLN4924 can potentiate many cell responses induced by chemotherapy drugs and radiation, including DNA damage, cell cycle arrest, and apoptosis. Simultaneously, MLN4924 can also inhibit the formation of immunosuppressive microenvironments, thereby enhancing anti-cancer immune responses. These MLN4924-mediated effects can impact the response of cancer cells to various treatments, as their migratory state is correlated with treatment resistance. Therefore, MLN4924 is not only an anti-cancer drug but also an effective sensitizer of chemotherapy, radiotherapy, and immunotherapy [8,9,26,59]. 

Previous studies suggest that the upregulation of the DNA damage repair pathways contributes to the tolerance of cancer cells to chemotherapy [71]. Among these, nucleotide excision repair (NER) is responsible for removing the majority of lesions triggered by chemotherapy drugs like cisplatin [71]. It was found that NER was mediated by CRL4 [44]. Moreover, many non-Cullin substrates of neddylation were also involved in regulating this process [4]. Therefore, theoretically, MLN4924 could induce the recurrence of cellular responses that were triggered by chemotherapeutics but rescued by NER. Actually, the resensitization effects of MLN4924 on cisplatin were demonstrated in ovarian cancer [63], head and neck squamous [44,72], and malignant pleural mesothelioma [73]. 

Downstream of the neddylation pathway, the NF-κB signaling pathway in cancer cells also contributes to the chemotherapy resistance of cancer cells [48], because it widely drives the transcription of multiple anti-apoptotic proteins and proinflammatory factors [74]. MLN4924 can inhibit clonal amplification and prevent apoptosis escape caused by the NF-κB signaling pathway, thus restoring the sensitivity of cancer cells to chemotherapy [75]. Mechanistically, MLN4924 partially reverses the chemotherapy resistance of cancer cells via abrogating the degradation of the NF-κB inhibitor mediated by the neddylation pathway [44,64,75]. In addition, cancer cells require rapid proliferation to dominate in competing with normal cells for fuels, and DNA topoisomerase 1 (TOP1) plays a crucial role in making it. The TOP1-cleavage complex (TOP1-cc) stabilized by camptothecin (CPT) is adverse to the normal function of TOP1 but can be degraded by the CRL3-mediated neddylation–UPS pathway, thus eliciting cancer cell tolerance [76]. However, the combination of CPT with MLN4924 can restore TOP1-cc accumulation and enhance the replication stress and DNA damage in cancer cells [76]. 

Likewise, MLN4924 recovers the sensitivity of multiple cancer cells to radiotherapy, while normal cells remain scarcely affected [46,59]. The main mechanism by which MLN4924 potentiates radiation damage in cancer cells is through its robust ability to induce DNA re-replication and cell cycle arrest. MLN4924, as a radiosensitizer, has been identified in pancreatic cancer [46], melanoma [59], head and neck cancer [72], and breast cancer [77].

The activation of the neddylation pathway and downstream NF-κB signaling pathway in cancer cells or non-malignant cells facilitates immunosuppression and cancer progression. MLN4924 can enhance the immune response of cancer cells, thereby promoting the mobilization of immune cells, which is a key mechanism in immunotherapy [26]. For example, MLN4924 can enhance the recognition and killing of MM (multiple myeloma) cells by natural killer (NK) cells by blocking the CRL1β-Trcp-NF-κB axis. This is because the axis mediates the degradation of MICA/B, which is responsible for activating NK cells and enabling them to identify MM cells [43]. In addition, the NF-κB signaling pathway negatively regulates the biological functions of endogenous tumor necrosis factors (TNFs). Cancer cells develop tolerance to TNFs due to prolonged microenvironmental exposure to TNFs [26]. MLN4924 can, in a TNFR1-dependent manner, resensitize cell death stimulated by TNFs via stabilizing Iκ-B, which hinders the nuclear translocation and activation of NF-κb [26]. 

Because the neddylation pathway plays an important role in cancer resistance to chemotherapy, radiotherapy, and cancer-related immunosuppression, MLN4924 exerts resensitization effects on various anti-cancer treatments [8,9,26,59]. In turn, the resensitization capacities of MLN4924 make the combination of MLN4924 with chemotherapy drugs, radiation, or immunotherapy drugs a feasible strategy to improve the effectiveness of anti-cancer treatment. This reflects the major asset of MLN4924 as an anti-cancer drug [1].

## 4. Pro-Cancer Side Effects of MLN4924

Previous reports have shown that MLN4924 exhibits impressive anti-cancer activity in cancer cells, the TME, and tumor virus-infected cells. Surprisingly, recent research has revealed that it can foster cancer cell motility in many ways, including off-target effects [21]. 

Due to the all-line inhibition effects of MLN4924 on the neddylation pathway, pro-cancer factors such as PD-L1, ASCT2, and HIF-1α inevitably accumulate in cancer cells [12]. In addition, since the neddylation pathway is involved in maintaining normal immune cell functions and infiltration into cancer cells, MLN4924 may hinder immune surveillance [14,78]. In a word, the wide neddylation inhibition induced by MLN4924 can partially enhance the growth, proliferation, and migration of cancer cells, which contradicts its use as an anti-cancer drug.

### 4.1. MLN4924 Enhances Cancer Onset by Stabilizing CRL Pro-Cancer Factors

MLN4924 is a potent anti-cancer drug with well-tolerated toxicity resulting from its excellent selectivity to cancer cells for which the neddylation pathway is generally overexpressed [1,26,59,77]. However, perhaps the low cytotoxicity of MLN4924 makes it a screener that stimulates drug resistance in non-lethal doses and/or short-term contact. It was reported that low concentrations (≤0.1 μM) of MLN4924 can enhance tumor sphere formation, while high concentrations inhibit it [22]. Likewise, Chang et al. [25] found that MLN4924 concentrations have biphasic effects on regulating viral latency in KSHV+ primary effusion lymphoma cells, suggesting that tumor viruses might display opposite susceptibility to different MLN4924 doses. However, whether MLN4924 concentrations elicit double-edged effects on tumor virus-infected cells remains to be further determined.

**Figure 2:**
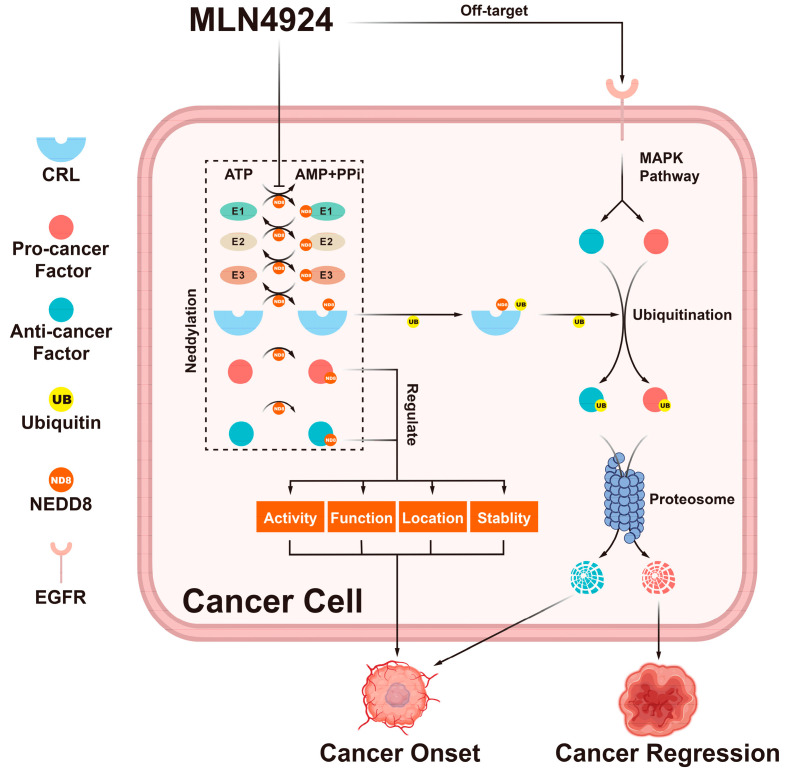
The double-edged effects of MLN4924. MLN4924 can block the entire neddylation pathway in cancer cells. This blockage can change the activity, function, location, and stability of neddylation substrates. Additionally, it can trigger the accumulation of CRL substrates. These factors regulated by the neddylation pathway can trigger many cellular responses that can either promote or inhibit tumor onset.

The double-edged effects of MLN4924 depend not only on concentrations but also on the substrates of the neddylation pathway, especially CRL substrates (Figure 2) [3,7,60]. Therefore, studying the mechanism of these side effects might shed light on improving drug combinations with MLN4924.

#### 4.1.1. c-Myc

c-Myc is a famous oncogenic protein and a substrate of CRL1FBXW7 [24]. Zhou et al. [22] found that within a certain range of 0–0.1 μM, MLN4924 upregulated c-Myc in cancer cells in a dose-dependent manner. At these low concentrations, MLN4924 can promote the proliferation, self-renewal, and differentiation of CSCs through c-Myc accumulation, which revealed MLN4924 pro-cancer side effects for the first time [22]. Furthermore, MLN4924-induced c-Myc accumulation can enhance the transcription of PD-L1, which is closely related to cancer cell immune escape [24]. Although c-Myc is a notorious oncogene protein, its accumulation was shown to promote the transcriptional activation of the pro-apoptotic protein NOXA, resulting in the apoptosis of head and neck squamous cell carcinoma (HNSCC) cells [9]. Ochiiwa et al. [79] reported that using TAS4464 (an NAE inhibitor), which is equipped with better selectivity than MLN4924, to treat acute myeloid leukemia (AML) could also cause c-Myc accumulation and the apoptosis of cancer cells to the same extent as MLN4924. It is clear that MLN4924 can cause an accumulation of c-Myc, which in turn leads to an increase in PD-L1 and/or NOXA expression. This suggests that it is important to assess the overall effectiveness of MLN4924 in treating cancer. The evaluation standard should be based on the balance between its anti-cancer and pro-cancer effects. 

#### 4.1.2. Programmed Death-Ligand (PD-L1)

In normal tissues, the immune checkpoints PD-1 and PD-L1 maintain immune homeostasis to prevent autoimmune diseases. However, this system can also facilitate tumor cell survival by activating tumor immune evasion [80,81,82]. The interaction between PD-1 and overexpressed PD-L1 on the surface of cancer cells can cause the inability, apoptosis, and exhaustion of T cells, which significantly impedes the process of immunotherapy. This suggests that an ideal anti-cancer drug should not interfere with tumor immune surveillance [23,24,82]. However, MLN4924 can, directly and indirectly, upregulate PD-L1 via multiple dimensions: (1) MLN4924 can promote the accumulation of PD-L1 by inhibiting CRL3SPOP and/or CRL1β-TRCP, which are its downstream substrates [78]; (2) the stabilization of PD-L1 transcriptional enhancers, such as c-Myc and HIF-1α, mediated by MLN4924 can indirectly increase its expression [24,82]; (3) and furthermore, it was discovered that MLN4924 acted as a signaling factor, inducing the MEK-JNK-AP1 (activator protein 1) axis [23]. AP1 subsequently binds to the enhancer of PD-L1 [22,23]. By contrast, the off-target effect of MLN4924 leads to a more significant increase in PD-L1 accumulation, suggesting its strong potential to trigger cancer-associated immunosuppression [23]. These findings seem to impinge on the application of MLN4924 as an immunotherapy sensitizer.

#### 4.1.3. Alanine–Serine–Cysteine Transporter 2 (ASCT2)

Glutamine plays a crucial role in cell metabolism. It can be converted into α-ketoglutaric acid, fueling cancer cell growth and proliferation. Therefore, the glutamine transporter ASCT2 is commonly overexpressed in different types of cancer cells [21]. Zhou et al. [21] verified that ASCT2, one of the CRL3SPOP substrates, was stabilized in breast cancer following MLN4924 treatment. This led to an increase in glutamine uptake and metabolism of cancer cells.

#### 4.1.4. Early Growth Response 1 (EGR1)

EGR1 is regulated by CRL1 [28], and its roles in cancer are cell-type-dependent [83]. EGR1 is considered to have a greater tendency to function as an anti-cancer factor. This is because the deletion of EGR1 has been linked to tumor onset [84]. Additionally, it can activate several anti-cancer factors, including p53 [85], PTEN [86], and TNFα [87]. However, EGR1 also plays pro-cancer roles in multiple gastrointestinal cancers [88]. EGR1 has also been found to be an oncogene which can promote prostate cancer metastasis [89]. As a substrate of CRL1, EGR1 can be stabilized in cancer cells following MLN4924 treatment [28]. Therefore, ERG1 accumulation triggered by MLN4924 can undoubtedly promote the spread of EGR1-promoted cancers mentioned above, which compromises its anti-cancer activity. This suggests that the side effects of MLN4924 may be cell-type-dependent.

#### 4.1.5. The Nuclear Factor-Erythroid 2 p45-Related Factor 2 (NRF2)

Whether NRF2 should be classified as an oncogene is still debated. In this review, we pay more attention to the pro-cancer side effects of NRF2. NRF2 is one of the CRL3 substrates [90,91] and its constitutive activation is correlated to cancer resistance to chemotherapy and radiotherapy, and poor prognosis [90]. It is theoretically impossible for NRF2 to accumulate in cancer cells due to the generally overexpressed neddylation pathway in these cells, which prevents NRF2 from exhibiting its carcinogenic characteristics. However, the activation of the NRF2 pathway commonly occurs in various cancers [90]. Mechanistically, either loss-of-function mutations in CRL3 that negatively regulate NRF2 expression or gain-of-function mutations in the NRF2-coding gene itself ease the paradox between the neddylation pathway and NRF2 activation [90,91]. Although NRF2 activation caused by the deletion of the neddylation pathway is relatively rare in cancers [91], recent research found that the neddylation defect induced by MLN4924 could cause the carcinogenic activation of the NRF2 pathway activation in melanoma [91], which had the same effect as the loss-of-function mutation of neddylation-associated genes.

#### 4.1.6. NF-κB-Inducing Kinase (NIK)

NIK regulates immunity and inflammation responses by activating the non-canonical NF-κB signaling pathway [47,92]. The unrestricted activation of NF-κB can cause autoimmune diseases and malignancies [92]. NIK was identified as a direct substrate of NEDD8, and neddylation could promote its degradation via UPS [6]. In addition, NIK was also negatively regulated by CRL4DCAF2 [92]. Therefore, the neddylation pathway maintains a low intracellular NIK level [5,92], which can be reversed by MLN4924 [6], causing the high expression or abnormal activation of NIK [93].

#### 4.1.7. Hypoxia-Inducible Factors-1α (HIF-1α)

When CRL2 is inactivated by MLN4924, it can lead to the stabilization of HIF-1α [69,94]. Therefore, MLN4924 was used to induce virus reactivation to treat EBV-positive cancer patients [56]. HIF-1α is primarily known as a cancer-associated factor. The pro-cancer effects of HIF-1α are characterized by promoting tumor angiogenesis and tumor-associated immune escape, inducing metabolic reprogramming and drug resistance [17]. So far, HIF-1α nuclear accumulation in cancer cells triggered by neddylation inhibition has been verified sufficiently [29,82]. HIF-1α is a major transcriptional activator of PD-L1, which could disable the antitumor activity of T cells [95]. The combination of an HIF-1α inhibitor and immune checkpoint blockade has been considered an effective strategy for immunotherapy [95]. Furthermore, the accumulation of HIF-1α caused by MLN4924 plays a crucial role in activating the REDD1/TSC1 axis [29,60]. This axis has the potential to suppress the activity of mTORC1, which is considered one of the factors that negatively regulate autophagy. As a result, protective autophagy is triggered in a mTORC1-dependent manner, which inhibits the apoptosis and senescence of cancer cells [29,60].

### 4.2. MLN4924 Supports the Formation of a Tumor-Comfortable Microenvironment

**Figure 3:**
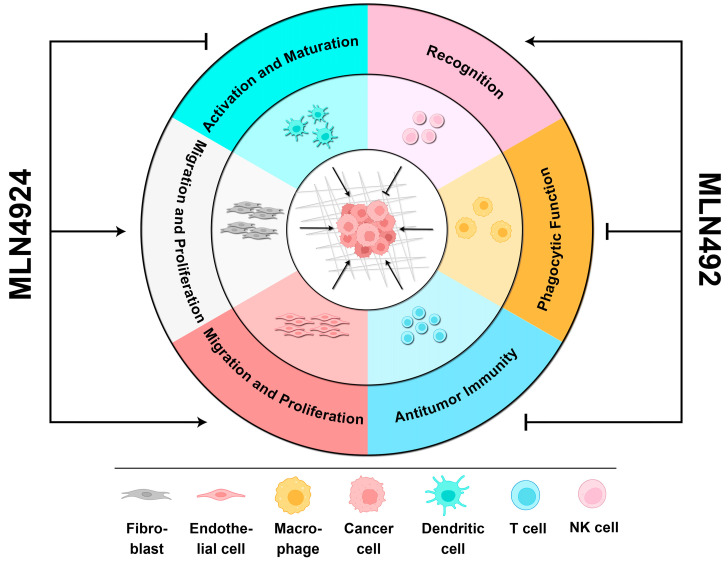
The biphasic roles of MLN4924 in the TME. When MLN4924 targets non-malignant cells within the TME, it can enhance cancer progression by (1) facilitating the migration and proliferation of fibroblasts and endothelial cells, (2) promoting the activation and maturation of dendritic cells, (3) and inhibiting the phagocytic function of macrophages and antitumor immunity mediated by T cells. However, it can also recover the recognition of cancer cells by NK cells to kill them.

MLN4924 targeting the neddylation pathway of non-malignant cells is also a double-edged sword, and its side effects can cause a favorable TME to promote cancer cell proliferation and invasion (Figure 3).

The neddylation pathways in T cells and DCs play a critical role in activating anti-cancer immunity [14]. However, the presence of MLN4924 could disrupt their maturation and antitumor functions, creating an immunosuppressive microenvironment [14,74,92]. Previous studies have suggested a positive correlation between the neddylation pathway and tumor growth and metastasis in TAMs, CAFs, and CAEs [14]. Therefore, inhibiting the neddylation pathway in non-malignant cells can effectively delay cancer progression [93]. However, a recent report verified that the neddylation pathway in macrophages helped recover their phagocytosis of cancer cells [30]. It seemed that the promotion of the neddylation pathway in macrophages, as related to the capacity to clear cancer cells, was partially reversed by MLN4924 [31]. Additionally, MLN4924 can aggravate chronic pancreatitis by upregulating the HIF-1α/CCL5 axis in pancreatic acinar cells, which is dependent on macrophage infiltration [94]. Chronic inflammation incubates a comfortable TME and harbors tumor cells. As mentioned above, tumor-infiltrating immune cells mainly mirror the pro-cancer side effects of MLN4924 on the TME. Further investigation is needed to determine how MLN4924 affects other non-immune cells within the TME.

The pro-cancer side effects of MLN4924 on the TME can also explain why it is necessary to evaluate the comprehensive anti-cancer efficacy of MLN4924 [14,52]. However, there has been insufficient focus on the correlation between the neddylation pathway and TME in recent years.

### 4.3. Rescue of MLN4924 Pro-Cancer Side Effects

It is unacceptable for an anti-cancer drug like MLN4924 to have pro-cancer side effects. Practically, the side effects of MLN4924 are a series of unexpected outcomes when used as a monotherapy. The practice of combining multiple drugs or therapeutic methods has been widely adopted and effectively applied in clinics. Therefore, whether to take advantage of the resensitization effects of MLN4924 or to avoid accompanying side effects, MLN4924 is suitable for this strategy [1]. 

Combining corresponding target inhibitors can significantly reduce the side effects of MLN4924 and prevent the accumulation of pro-cancer CRL substrates. For example, cooperating with MEK inhibitors or immune checkpoint inhibitors can rescue PD-L1 upregulation triggered by MLN4924 turning on the ERK1/2-JNK signaling pathway or directly inhibiting CRL1FBXW7/CRL3SPOP-mediated UPS [10,23,24]. For ASCT2 accumulation induced by CRL3SPOP inhibition, adding its inhibitor V-9302 can effectively reverse the enhancements of MLN4924 to glutamine metabolism of cancer cells [21]. Xu et al. [6] discovered that inhibiting NIK with B022 can reduce the abnormal NIK signal triggered by MLN4924. Zhao et al. [29] suggested that MLN4924 combined with an autophagy inhibitor could potentially suppress protective autophagy and trigger apoptosis in cancer cells. Actually, in current clinical trials, MLN4924 plus many other anti-cancer treatments have been scheduled in order to assess the combinations of side effects, efficiency, safety, and tolerability (Table 2). In addition, developing a cancer-target delivery vehicle for MLN4924 can avoid the formation of a pro-cancer microenvironment caused by MLN4924 targeting non-malignant cells in the TME [14]. However, owing to the double-edged effects of MLN4924 on the TME, this strategy neglected and abandoned the anti-cancer effects induced by MLN4924 on non-malignant cells. However, it is not a sustainable solution to avoid the side effects of MLN4924 by utilizing combination therapy. Developing a proper vehicle for MLN4924 also faces multiple challenges, especially biocompatibility. Therefore, continuously developing more efficient drugs and selective drugs with appropriate targets is a long-term pursuit in the field of anti-cancer treatment (Table 3).

## 5. Perspectives

Besides MLN4924, many drugs targeting the neddylation pathway are in development or have been developed for clinical use (Table 3) [1]. In this review, we highlighted the double-edged effects of the neddylation pathway on cancer cell behavior (Figure 2), explaining why the side effects of MLN4924 cannot be solely attributed to off-target effects [21]. Although the side effects caused by MLN4924 can be partially alleviated by combining it with other anti-cancer treatments (Table 2), it is indicated that the all-line blockage of the neddylation pathway triggered by MLN4924, which causes MLN4924 to oscillate between anti-cancer and pro-cancer effects, seems excessive.

Therefore, novel requirements have been proposed for developing next-generation drugs that target the neddylation pathway [1]. Previous research has suggested that inhibitors targeting downstream enzymes of neddylation may have higher specificity and selectivity than NAE inhibitors with fewer pro-cancer side effects [1,43,97,100]. For example, the small-molecule inhibitor HA-9104 targeting UBE2F selectively inactivates CRL5 and can inhibit lung cancer progression by inducing the accumulation of the pro-apoptotic protein NOXA [100]. The inhibitor of UBE2M involved in the neddylation of Cullin1-4, arctigenin, effectively inhibited the malignant phenotype of cancer cells [99]. The DCN1-UBE2M complex inhibitor NAcM-OPT can also selectively inhibit CRL1 and CRL3. This resulted in the upgradation of MICA/B on the surface of MM cells, similar to MLN4924, promoting the recognition and killing of MM cells by NK cells [43]. The above cases can briefly provide new threads for next-generation inhibitors targeting the neddylation pathway and novel strategies for patients with cancer recurrence following MLN4924 treatment.

## Figures and Tables

**Table 2:** The combinations of MLN4924 in current clinical trials.

Combination Treatment	Target Cancer	Clinical Status	References
Paclitaxel/Gemcitabine/Docetaxel/Carboplatin	Solid Cancers	Phase I	https://clinicaltrials.gov/study/NCT01862328?cond=Cancer&intr=MLN4924&rank=4&tab=table (accessed on 10 June 2024)
Azacitidine	AML	Phase I	https://clinicaltrials.gov/study/NCT01814826?cond=Cancer&intr=MLN4924&rank=5 (accessed on 10 June 2024)
Pembrolizumab	dMMR/MSI-H Cancers	Phase I/II	https://clinicaltrials.gov/study/NCT04800627?cond=Cancer&intr=MLN4924&rank=8 (accessed on 10 June 2024)
Irinotecan/Temozolomide	Solid Cancers or Lymphoma	Phase I	https://clinicaltrials.gov/study/NCT03323034?cond=Cancer&intr=MLN4924&rank=9 (accessed on 10 June 2024)
Belinostat	AML	Phase I	https://clinicaltrials.gov/study/NCT03772925?cond=Cancer&intr=MLN4924&rank=10 (accessed on 10 June 2024)
Azacitidine	AML	Phase II	https://clinicaltrials.gov/study/NCT03745352?cond=Cancer&intr=MLN4924&page=2&rank=11 (accessed on 10 June 2024)
Carboplatin/Paclitaxel	Non-small-Cell Lung Cancer (NSCLC)	Phase II	https://clinicaltrials.gov/study/NCT03965689?cond=Cancer&intr=MLN4924&page=2&rank=12 (accessed on 10 June 2024)
Ixazomib	MM	Phase I	https://clinicaltrials.gov/study/NCT03770260?cond=Cancer&intr=MLN4924&page=2&rank=13 (accessed on 10 June 2024)
Etoposide/Prednisone	Diffuse Large B-Cell Lymphoma	Phase I/II	https://clinicaltrials.gov/study/NCT01415765?cond=Cancer&intr=MLN4924&page=2&rank=14 (accessed on 10 June 2024)
Azacitidine/Venetoclax	AML	Phase I/II	https://clinicaltrials.gov/study/NCT03862157?cond=Cancer&intr=MLN4924&page=2&rank=15 (accessed on 10 June 2024)
Ibrutinib	Chronic Lymphocytic Leukemia (CLL) or AML	Phase I	https://clinicaltrials.gov/study/NCT03479268?cond=Cancer&intr=MLN4924&page=2&rank=18 (accessed on 10 June 2024)
Carboplatin/Paclitaxel	Cholangiocarcinoma	Phase II	https://clinicaltrials.gov/study/NCT04175912?cond=Cancer&intr=MLN4924&page=2&rank=19 (accessed on 10 June 2024)
Cytarabine/Idarubicin	AML	Phase I/II	https://clinicaltrials.gov/study/NCT03330821?cond=Cancer&intr=MLN4924&page=3&rank=28 (accessed on 10 June 2024)
Fludarabine Phosphate	AML	Phase I	https://clinicaltrials.gov/study/NCT03813147?cond=Cancer&intr=MLN4924&page=3&rank=29 (accessed on 10 June 2024)
Docetaxel	NSCLC	Phase II	https://clinicaltrials.gov/study/NCT03228186?cond=Cancer&intr=MLN4924&page=4&rank=31 (accessed on 10 June 2024)
Vincristine/Dexamethasone	Acute Lymphoblastic Leukemia	Phase I	https://clinicaltrials.gov/study/NCT03349281?cond=Cancer&intr=MLN4924&page=4&rank=32 (accessed on 10 June 2024)
Pemetrexed/Cisplatin	Mesothelioma	Phase II	https://clinicaltrials.gov/study/NCT03319537?cond=Cancer&intr=MLN4924&page=4&rank=40 (accessed on 10 June 2024)

**Table 3:** Neddylation inhibitors.

Neddylation Inhibitor	Target	Clinical Status	References
MLN4924	NAE	Phase I/II/III	[16]
TAS4464	NAE	Phase I	[79]
ZM223	NAE	N/A	[96]
DC-2	UBE2M	Phase I/II	[97]
DI-591	UBE2M	N/A	[97]
DI-404	UBE2M	N/A	[97]
DI-1859	UBE2M	N/A	[98]
WS-383	UBE2M	N/A	[97]
Arctigenin	UBE2M	Phase I	[99]
NAcM-OPT	UBE2M	N/A	[43]
NAcM-HIT	UBE2M	N/A	[97]
NAcM-COV	UBE2M	N/A	[97]
HA-9104	UBE2F	N/A	[100]
Gossypol	RBX1/RBX2	Phase II/III/IV	[5]

## Data Availability

Not applicable.
